# Endometrial Stem Cell Markers: Current Concepts and Unresolved Questions

**DOI:** 10.3390/ijms19103240

**Published:** 2018-10-19

**Authors:** Nicola Tempest, Alison Maclean, Dharani K. Hapangama

**Affiliations:** 1 Liverpool Women’s Hospital NHS Foundation Trust, Liverpool L8 7SS, UK; ntempest@liverpool.ac.uk (N.T.); alisonm2504@gmail.com (A.M.); 2 Department of Women’s and Children’s Health, Institute of Translational Medicine, University of Liverpool, Liverpool L8 7SS, UK

**Keywords:** endometrium, adult stem cells, endometrial regeneration, stem cell markers, endometriosis, endometrial cancer

## Abstract

The human endometrium is a highly regenerative organ undergoing over 400 cycles of shedding and regeneration over a woman’s lifetime. Menstrual shedding and the subsequent repair of the functional layer of the endometrium is a process unique to humans and higher-order primates. This massive regenerative capacity is thought to have a stem cell basis, with human endometrial stromal stem cells having already been extensively studied. Studies on endometrial epithelial stem cells are sparse, and the current belief is that the endometrial epithelial stem cells reside in the terminal ends of the basalis glands at the endometrial/myometrial interface. Since almost all endometrial pathologies are thought to originate from aberrations in stem cells that regularly regenerate the functionalis layer, expansion of our current understanding of stem cells is necessary in order for curative treatment strategies to be developed. This review critically appraises the postulated markers in order to identify endometrial stem cells. It also examines the current evidence supporting the existence of epithelial stem cells in the human endometrium that are likely to be involved both in glandular regeneration and in the pathogenesis of endometrial proliferative diseases such as endometriosis and endometrial cancer.

## 1. Introduction

**Figure 1:**
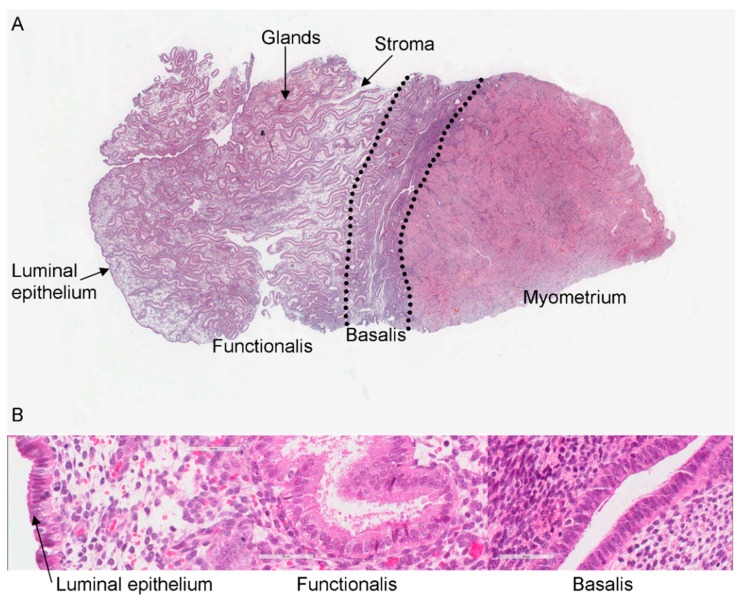
(**A**): Low power (100×) micrograph of full thickness biopsy of human endometrium and sub-endometrial myometrium stained with Hematoxylin and Eosin. (**B**): Representative micrograph depicting the distinct anatomical areas in the human endometrium, luminal epithelium, functionalis, and basalis (magnification 400×).

The human endometrium is a highly regenerative organ undergoing over 400 cycles of shedding and regeneration over a woman’s life time [1,2,3]. Menstrual shedding, and the subsequent repair of the endometrial functionalis, is a process unique to humans and higher-order primates [4,5] (Figure 1A,B). The endometrium re-grows from a mere 1–2 mm thickness after menstrual shedding to 14 mm thickness in the secretory phase of the menstrual cycle [6], and is able to completely regenerate after parturition, and in post-menopausal (PM) women when exposed to oestrogen replacement therapy [2,7]. Even after extensive iatrogenic destructive procedures such as ablation [8], the endometrium regrows in some women who continue to bleed (25–75%) [9,10]. This huge regenerative ability suggests that the endometrium has a stem cell basis that supports the tissue maintenance/regrowth.

**Figure 2:**
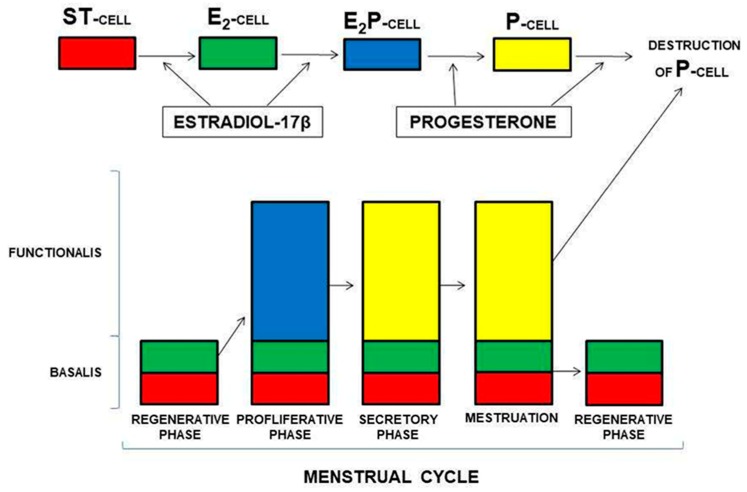
Hormone dependent regulation of the ratio of cell types in the endometrium (Abbreviations ST-cell—stem cell, E2-cell—oestrogen sensitive cell, E2P-cell—oestrogen-progesterone sensitive cell, P-cell—progesterone sensitive cell). Figure adapted from Reference [11].

Prianishnikov was the first to consider the existence of endometrial adult stem cells (ASCs) and, in 1978, he proposed ASCs to reside in the deeper basalis layer, with their differentiation marked by functional changes (acquiring) in hormonal receptivity [11]. He suggested a hierarchical hormone receptiveness in endometrial cells, matching their level of maturity, and therefore, the most primitive hormone-independent ASCs initially differentiate first into oestrogen-dependent cells, and then they may further differentiate into both oestrogen and progesterone-dependent cells. Terminally-differentiated cells were expected to be only progesterone-dependent, and were postulated to have a limited lifespan [11] (Figure 2).

This hypothesis proposing that the human endometrium regenerates from the deeper basalis layer (which is the proposed germinal compartment that persists after menstruation and is responsible for the regeneration of the new upper/superficial functionalis layer) has been echoed many times [12,13,14,15].

Identifying human endometrial epithelial stem cells is problematic due to the lack of specific markers for isolating and examining them for functional properties [16]. Endometrial epithelial cells in particular are difficult to culture in vitro for long durations, with the in vivo phenotype of these cells not maintained using traditional 2D culture methods. For this reason, functional assays that have been developed to examine the stem cell properties in vitro, may not be suitable for endometrial epithelial cells. Furthermore, the true and conclusive confirmation of an endometrial epithelial stem cell requires the demonstration that they are able to produce all of the epithelial cell types that exist in all regions of the endometrium. However, the characterisation of all different endometrial epithelial cell subtypes, to ascertain the mature progeny of the putative stem cell, is not yet complete in the human endometrium.

Early work on endometrial regeneration has been gleaned through scanning electron microscopy (SEM) studies, this includes some evidence for the existence of endometrial epithelial stem cells. SEM studies have confirmed that the total regeneration time for postmenstrual surface re-epithelialisation is approximately 48 h [17] (with the regenerative period beginning between cycle days 2 and 3, and ending between cycle days 4 and 5). This time interval coincides with the maximum area of the denuded basalis [17]. The SEM data also suggests that the surface epithelial repair occurs by simultaneous and progressive outgrowth from the remaining stumps (“mouths”) of basal glands, and by outgrowth from the surface epithelium, adjacent to the denuded areas, that has not been lost from the isthmic and cornual regions [17]. A similar observation was reported in rabbit endometrium; the newly restored surface lining was derived from the intact surface epithelium that remained bordering the artificially (rabbits do not menstruate) denuded area [18]. The other important findings of the SEM studies are the descriptions of morphological differences in endometrial epithelial subtypes. For example, ciliated and non-ciliated cells have been observed in the luminal epithelium (LE) [19]. Cells with the same morphology as that of endocrine cells were found in the lower layers of the epithelium at late gestation (clear cells) [20], and are morphologically similar to endocrine cells in other tissues [21]. This further confirms the existence of different epithelial subtypes that are yet to be characterised for the expression of distinct markers or for their possible functional diversity.

## 2. Scope of This Review

In this review, we will examine the evidence for particular markers to delineate the endometrial stem cell population. We will describe the available evidence under the three main hypotheses that stem cell marker identification in the endometrium has been undertaken, by:

Identifying markers highly expressed in cells with some in vitro stem cell properties (e.g., clonogenic cells, side population (SP) cells).

Identifying markers expressed by cells located in the postulated stem cell niche (basalis and PM glandular epithelium).

Examining endometrium for the expression of putative stem cell markers identified to be expressed in epithelial stem cells (ESCs), or other stem cells of different tissues (e.g., OCT4, Mushashi-1, LGR5, Notch1/Numb).

## 3. Identifying Markers That Are Highly Expressed in Cells with Some In Vitro Stem Cell Properties

Stromal ASC work was initiated with identifying markers preferentially expressed in the cell populations demonstrating higher clonogenic properties in vitro, by Gargett and colleagues [12]. The in vitro colony-forming efficiency assay they used is thought to be a method that enriches stem cells by inoculating single cell suspensions derived from freshly harvested tissue at low density, in order to generate colonies from individual cells.

Endometrial stromal ASCs were also shown to be capable of multi-lineage differentiation into fat, bone (confirmed with presence of osteopontin, ostenectin and alkaline phosphatase) [22], cartilage, skeletal muscle [23,24], and smooth muscle (expressing specific smooth muscle cell markers including alpha-smooth muscle actin (α-SMA), desmin, vinculin and calponin) [25]. Plasticity has also been shown by the trans-differentiation of endometrial stromal ASCs into neural (neural and glial lineage markers such as Nestin, NF-L, MAP2, PDGFRa, CNP, Olig2, MBP and GFAP) [24,26], Schwann cells (expression of S100 and P75 noted) [27], Oligodendrocytes [28], pancreatic cells (shown by secretion of insulin and markers of β cells such as PDX1, proinsulin and c-peptide) [29,30], urinary bladder epithelial cells (urothelium, as tested by urothelium-specific genes and proteins, uroplakin-Ia/Ib, II, III and cytokeratin 20) [31], hepatocytes (biomarkers albumin and cytokeratin 8, reduced α-fetoprotein and α-SMA expression, synthesised urea, and stored glycogen) [32,33], and megakaryocytes (identified by expression of CD41a and CD42b and reduction of pluripotent transcription factors Oct4 and Sox2, platelets were seen as functional as evidenced by the upregulation of CD62p expression and fibrinogen binding following thrombin stimulation), both in vitro and in animal models [34,35].

Studies employing animal models of Duchenne muscular dystrophy [36], stroke [37], diabetes [38], Parkinson’s disease [39,40], and critical limb ischemia [41] have suggested that endometrial stromal ASCs improve outcomes, postulating an in vivo differentiation potential for these cells.

Further in vitro stem cell properties that were utilised to identify endometrial ASCs include examining the side population (SP) cells and label retaining cells (LRC) as detailed below.

### 3.1. Markers Identified in Clonogenic Cells

#### 3.1.1. CD146 and Platelet Derived Growth Factor–Receptor β Co-Expression

The first markers proposed to identify an endometrial stromal ASC population were CD146 and platelet-derived growth factor–receptor β (PDGF-Rβ) (2 perivascular cell markers) [22]. This is because their co-expression was detected in cells with higher clonogenic ability in vitro. These cells were located in the perivascular area in both the functionalis and the basalis of the intact full thickness human endometrium [42]. In vitro FACS sorted CD146^+^/PDGF-Rβ^+^ cells had significantly greater colony-forming capabilities than CD146^−^/PDGF-Rβ^−^ cell populations (7.7 ± 1.7% versus 0.7 ± 0.2% respectively) [42]. CD146^+^/PDGF-Rβ^+^ cells produced more large colonies with densely packed cells and a high nuclear:cytoplasmic ratio. The CD146^+^PDGF-Rβ^+^ cells expressed typical mesenchymal stem cell (MSC) surface markers such as CD29, CD44, CD73, CD90 and CD105, and were negative for haematopoietic (CD34, CD45) and endothelial markers (CD31) [42]. When cultured in appropriate induction media, the CD146^+^PDGF-Rβ^+^ cells underwent multi-lineage mesenchymal differentiation into adipogenic, myogenic, chondrogenic, and osteoblastic lineages [42]. However, these studies used pooled, clonally-derived CD146^+^PDGF-Rβ^+^ cells (not singly expanded clones) and did not use positive control cells with known multi-lineage differentiation potential (such as human mesenchymal stem cells (hMSCs)) to determine their true differentiation ability. Apart from the colony-forming capacity, and multi-lineage differentiation ability, the authors did not examine other stem cell-related features and functions of the CD146^+^PDGF-Rβ^+^ cells. Therefore, we are not able to comment on their capacity to produce endometrial stroma either in a more physiologically relevant 3D culture in vitro, or their in vivo tissue reconstitution ability. Therefore, it is difficult to decisively conclude that these cells are an ASC population that regenerates the endometrial stroma. However, it is thought that the CD146^+^PDGF-Rβ^+^ subpopulation are similar to bone marrow and adipose tissue MSCs in their differentiation potential, and their perivascular location is also shared by MSCs in many other organs [43].

#### 3.1.2. SUSD2

Sushi domain containing-2^+^ (SUSD2^+^) (also known as W5C5) was the next endometrial stromal ASC marker to be reported and it was particularly successful in selecting endometrial MSCs [44]. SUSD2^+^ cells represented 4.2 ± 0.6% of the freshly sorted endometrial stromal cells using flow cytometry, and assumed a peri-vascular location both in the basalis, and functionalis, with a significantly greater clonogenicity (median 3.6: range, 0.7–6.9) than depleted counterparts (median 0.6: range, 0.1–3.8). W5C5^+^ cells were able to be differentiated into adipocytes, osteocytes, chondrocytes, myocytes, and endothelial cells (no MSCs were used as an external control), producing endometrial stromal-like tissue in vivo. SUSD2^+^ cells were transplanted under the kidney capsule of non-obese diabetic, severe combined immunodeficiency mutation and interleukin-2Rγ allelic mutation (NSG) mice, and white growths (small masses) were identified macroscopically on 2 out of 10 kidneys. When the mice were examined by histological and microscopic analyses, stromal-like connective tissue was revealed under all of the kidney capsules. The SUSD2^+^ cells produced significantly greater numbers of CFUs and the study identified SUSD2^+^ as a single marker capable of purifying endometrial MSCs [44], thus negating the need to use two markers (CD146^+^/PDGF-Rβ^+^) that were proposed in the earlier studies. Although there was a considerable overlap of SUSD2 expression with CD146^+^PDGF-Rβ^+^ expression, the co-expression of the markers was not consistent. Moreover, endometrial pericytes are postulated to express CD146^+^/PDGF-Rβ^+^ whilst perivascular location is proposed for the SUSD2^+^ cells suggesting some dissimilarities between the cells identified by these markers [15]. 

### 3.2. Side Population Cells

SP analysis distinguishes stem and progenitor cells from other more differentiated, somatic cells based on their ability to extrude DNA-binding fluorescent dyes (such as Hoechst 33342, a fluorescent dye that binds to the minor groove of deoxyribonucleic acid) since they express special ATP-binding cassette–containing pumps (ABC transporters—a type of membrane-bound active transporter, ABCG2). SP cells (0–5%) have been identified in fresh isolates [45,46,47] and short-term cultures [48] of human endometrial cells. The percentage of SP cells in single cell endometrial suspensions (derived from different patient samples) was reported to be highly variable between individuals. However, higher numbers seemed to be found in the menstrual [48] and proliferative [45,47] stages of the cycle. This variability was also mirrored by CFU activity in human endometrium. There is no consensus yet on whether the absolute number of SP cells are stable throughout the menstrual cycle or not. In agreement with the hypothesis that ASCs constitute a small, quiescent and static resident population [47], the decline of SPs in the secretory phase may result from dilution as the functionalis grows and increases in thickness. When SP cells (freshly sorted) showed little growth in culture, the authors argued that this was secondary to them being quiescent (most of the cells (85%), being in G0 phase of the cell cycle). This is a proposed feature of ASCs, but, in contrast, the SP cells sorted from endometrial short-term cultures were, primarily, in G1 and G1/M/S phases [45]. Endometrial SP cells sorted from short-term cultures did not express endometrial epithelial (CD9), or stromal (CD13) cell differentiation markers to start with, but these markers were re-expressed in subsequent long-term Matrigel cultures, indicating a capacity to differentiate into CD9^+^E-cadherin^+^ gland-like organoids (suggesting epithelial differentiation) and CD13^+^ stromal clusters when cultured for a further 2 months on collagen-coated dishes [48]. However, even FACS, the most efficient sorting method available, does not have 100% efficiency/purity in sorting these cells. Therefore, particularly in long-term culture, a small percentage of both epithelial and stromal cells that are likely to have been contaminating the initial SP (although the authors reported that they do not express measurable levels of epithelial/stromal markers) may have expanded. The other reports examining the endometrial SP cells show SP cells in both the stromal and epithelial populations [1,47,49]. Furthermore, it is known that endometrial cells in 2D culture undergo culture-related changes and loss of cellular phenotypical markers and thus, cultured cells may lose their markers, but they have the capacity to regain them in a more physiologically relevant growth environment, the 3D matrix. Finally, the achievement of a single cell suspension from solid tissue samples (necessary for cell sorting (e.g., enzymatic digestion)) will remove the cell surface proteins, which will later be re-formed in cells with prolonged culture. All these issues need to be considered when interpreting the current literature. The sorted short-term cultured SP cells were able to be maintained in culture for 3 months, whereas the non-SP cells sorted from the short-term cultured SP cells became senescent within 3 months [48]. This evidence concurs with the longevity associated with ASCs as opposed to differentiated cells.

To demonstrate their functional potential, cultured endometrial SP cells were shown to decidualise after being treated with oestradiol and progesterone, therefore confirming their ability to assume the morphologic, functional, and known terminal differentiation changes characteristic of the secretory endometrium [45].

Additionally, by differentiating into adipocytes and osteoblasts in vitro, endometrial epithelial SP cells have been shown to be multipotent, however, once again the differentiation potential was not compared with a control stem cell type such as hMSCs [1]. After 2 weeks in culture, the authors reported the presence of Oil Red O lipid vacuoles in adipogenic induction media (but the round morphology typical of adipocytes was not seen). Similarly, in osteogenic induction media, positive immuno-reactivity for bone sialoprotein was reported. This evidence is intriguing and needs to be considered with the possibility of contamination with stromal cells (which are known to have the ability to differentiate in the mesenchymal lineages). Further evidence has been produced by Kurita et al., with their elegant set of xenograft experiments demonstrating the adult endometrial epithelium to be lineage specific [50].

Masuda et al., demonstrated unipotency of the endometrial epithelial SP cells in vitro by injecting them under the mouse kidney capsule (mesodermal derivative), and generating endometrial tissue; those cells did not differentiate into kidney parenchyma [44]. This unipotency was echoed with work showing that SSEA-1 positive endometrial cells grown in a chimeric explant model, using kidney tissue isolated from CD1 neonatal mice, injected under the kidney capsule, produced endometrial gland like structures staining positive for endometrial differentiation markers [51].

Serum oestradiol levels have been shown to change in the same manner as the proportion of SP cells in postpartum mice, perhaps indicating that oestrogen is a prerequisite for increasing the size of SP cells populations [52]. This finding of increased oestrogen was confirmed within a mouse endometrial injury model; stromal SP cells significantly increased 6 h after injury, but they were dependent on oestrogen, not progesterone nor a combination of oestrogen and progesterone [53].

A relationship between stromal and epithelial ASCs was seen when stromal SP cells were transplanted under the kidney capsule in mice; only endometrial stroma was formed. When epithelial SP cells were transplanted, only epithelium was formed. Endometrial-like tissue was only generated when both these populations were combined. This observation would support the existence of two distinct ASCs—a stromal and an epithelial ASC [49].

### 3.3. Markers Identified in Cells with Other MSC Properties

#### 3.3.1. Menstrual Blood-Derived Stem Cells

Another functional assay to identify stem cells is their ability to differentiate in to a variety of cell types, i.e., the differentiation potential. In 2007, it was reported that menstrual blood could be used to obtain endometrial stromal ASCs, and these were capable of differentiation into adipocytes, osteoblasts, chondrocytes, cardiocytes, myocytes, and endothelia [54]. These menstrual blood-derived stromal ASCs (mbdASCs) were also capable of trans-differentiation into endodermal and ectodermal tissue, such as hepatocytes, pulmonary epithelia and neurones. The mbdASCs were mononuclear, and demonstrated positive immuno-reactivity for CD90, CD73, and CD103, but were devoid of CD34 and CD45, suggesting the cells are of mesenchymal, not haematopoietic in origin [54]. The differentiation potential of these cells was compared to cord blood-derived MSCs, by comparing the expression of proteins related to stem cell function. Matrix metalloproteases (MMP-3 and MMP-10), cytokine growth factors (GM-CSF, PDGF-BB) and angiogenic factors (ANG-2) were shown to be expressed by the mbdASCs at higher levels than the cord blood-derived MSCs, suggesting that the mbdASCs have similar phenotypical features to ASCs, but differences in their function exist. mbdASCs are an attractive target as a treatment for many diseases, due to easy access, non-invasive collection and, if there is a potential therapeutic use, their possible autologous utility will deem them superior to many other ASC types [55]. The use of autologous cells for the subacute phase of stroke offers a practical clinical application [56]. When grown in appropriate conditioned media, the mbdASCs express neuronal phenotypic markers (Nestin, MAP2), and in an in vitro stroke model of oxygen and glucose deprivation it was found that oxygen and glucose deprived-exposed primary rat neurons, (co-cultured with mbdASCs or exposed to the media collected from cultured menstrual blood), exhibited significantly reduced cell death [55]. Transplantation of mbdASCs (either intra-cerebrally, or intravenously, and without immunosuppression) into a rat model of ischaemic stroke, significantly reduced behavioural and histological impairments, compared to vehicle-infused rats, supporting the use of mbdASCs as a stem cell source for cell therapy in stroke [55,56,57,58] and other basal ganglia disorders, such as Parkinson’s and Huntington’s disease [59].

Sepsis (in the cecal ligation and puncture mouse model) has also been shown to have improved outcomes when mbdASCs are utilised in the treatment regime, alongside antibiotics. mbdASCs, in synergy with antibiotics, improved the survival rate (95%) in comparison; with saline (6%); antibiotics alone (73%); and mbdASCs alone (48%); concluding that mbdASCs could constitute a feasible approach for the future clinical treatment of sepsis [60].

A mouse model of premature ovarian failure, treated with mbdASCs, expressed higher levels of ovarian markers (AMH, inhibin α/β and FSH receptor), and the proliferative marker Ki67. In addition, the overall weight, plasma oestrogen level, and number of normal follicles increased overtime compared with controls [61].

mbdASCs have been differentiated into hepatocyte-like cells, and demonstrated in vitro mature hepatocyte functions such as urea synthesis, glycogen storage, and indocyanine green uptake; showing their potential to be used in chronic liver disease management [62,63,64].

The type 1 diabetes mellitus mouse model was used to show the therapeutic effects of mbdASCs on the mechanism of β-cell regeneration after transplantation [38]. The mbdASCs reversed hyperglycaemia and weight loss, prolonged lifespan, and increased insulin production in the diabetic mice. The mice recovered islet structures and increased their β-cell number, with the majority of the mbdASCs migrating into the damaged pancreas, and being located at the islet, duct, and exocrine tissue. The mbdASCs were found to enhance neurogenin3 expression (represents endocrine progenitors) rather than differentiate into insulin-producing cells, concluding that they stimulated β-cell regeneration through promoting differentiation of endogenous progenitor cells [38].

mbdASCs have also been proposed to be used for bone tissue-engineering purposes (taking advantage of their osteogenic driving potential) [65]; dermatological lesions and diseases [66]; heart muscle repair [67]; limb ischaemia [41]; and muscular dystrophy [68].

More recently, it has been shown that these mbdASCs have the ability to secrete decidualisation markers (prolactin and insulin-like growth factor binding protein-1), and differentiate into decidualised cells, leading to the potential of a therapy for decidualisation insufficiency [69]. When cultured in differentiation-inducing media supplemented with 20% human follicular fluid, the human mbdASCs form oocyte-like cells and express germ cell markers. Cells also expressed FSH and LH receptors, and produced oestrogen and progesterone regulated by gonadotrophin, suggesting a potential to differentiate in to ovarian tissue [70]. The exact origin of these cells (endometrial or bone marrow derived), however, is not known. Another further important consideration is that most diseases for which stem cell therapy has been proposed (e.g., prolapses, strokes, myocardial infarctions) occur in older, PM women who do not menstruate. The regenerative ability of the PM endometrium may allow stem cell harvesting after hormone treatment, yet the effect of such a treatment can be detrimental on the health of an elderly patient with cardiovascular disease and other comorbidities. Therefore, these practical challenges need to be considered when pursuing menstrual blood as a source of stem cells for autologous therapeutic avenues.

#### 3.3.2. Bone Marrow as a Source of Endometrial ASCs

Bone marrow, as a source of endometrial regeneration, is supported by the ability of bone marrow-derived MSC to produce ‘decidua-like’ stroma, after activation of the protein kinase cAMP-dependent pathway in vitro [71]; together with bone marrow-derived cells being found in the decidua of normal murine pregnancy [72]. Stem cells of bone marrow origin typically express markers such as CD34. Co-culture of bone marrow-derived cells with endometrial stromal cells, and oestrogen stimulation, result in their differentiation into CK^+^ endometrial epithelial-like cells [73].

When male bone marrow-derived cells were transplanted into female mice, Fluorescence in situ hybridisation demonstrated the Y-chromosomes to be present only in 0.0002% of CD45-/F4/80- epithelial cells, and 0.0003% of CD45-/F4/80- stromal cells of the endometrium [74]. In another set of experiments, samples from female mice harvested 40 days after a haematological stem cell transplant, showed an average of 6% donor-derived cells in the endometrium, concluding that bone marrow-derived endothelial progenitors contribute to the formation of new blood vessels in the endometrium [75].

In a study of Human leucocyte antigen mismatch transplants, donor-derived endometrial cells were detected in endometrial biopsy samples from all bone marrow recipients, and accounted for a wide range (0.2 to 48%) of epithelial (displaying CD9 marker), and vimentin positive stromal cells (0.3 to 52%) [76,77,78]. SP cells were not shown to be formed by XY donor-derived cells [77].

Therefore, the evidence presented above may suggest bone marrow to also be a source of stem cells for endometrial regeneration, but its contribution seems to be low. It is likely that bone marrow could be implicated in endometrial repair after times of massive injury, such as ablation, and in the formation of the decidua; when the endometrium requires ‘extra-assistance’.

In a more recent study, authors using chimeric mouse endometrial tissue reconstitution with bone marrow derived from transgenic *mTert*-green fluorescent protein (GFP) reporter mice and irradiated recipients have suggested that bone marrow stem cells do not contribute to any of the endometrial cell lineages such as stroma, epithelium or endothelium [79]. All of the cells that were detected in the endometrium were immune cells expressing the pan-leukocyte marker CD45, including CD3^+^ T cells and F4/80^+^ macrophages; that immuno-stained weakly for CD45. The macrophages were abundant in the stroma, infiltrating the epithelial and vascular compartments, and it was noted that they could easily be mistaken for bone marrow-derived endometrial cells. The authors concluded (in disagreement with previous studies) that bone marrow cells are unlikely to transdifferentiate into endometrial stroma, epithelium and endothelium. They warned of the massive implications, since bone marrow-derived endometrial stem cells have been anticipated to be useful for numerous treatment strategies discussed previously [79]. Further work to clarify this possibility is urgently needed before their clinical applications.

### 3.4. Label Retaining Cells

Locating LRC in animals (the use of the LRC technique in humans is not permitted due to BrdU being a recognised health hazard) is a method of identifying somatic stem/progenitor cells and their location in the stem cell niche, when specific markers are unknown. This method relies on the infrequent cell turnover of most ASCs, in comparison to rapidly proliferating TA cells [2,80]. Mouse endometrium was pulse labelled with BrdU and studied after an 8-week chase to identify endometrial LRC.

#### 3.4.1. Epithelial LRCs

Gargett’s group reported that 3% of the epithelial nuclei were BrdU^+^ and were located in the LE. They were shown not to express ERα through dual labelling IF, providing evidence that LE stem/progenitor cells are responsible for the growth of glands during development and in cycling mice [80]. In ovariectomised prepubertal mice, the first cells to proliferate in oestrogen-stimulated endometrial growth differed from ovariectomised cycling mice, in the first, the epithelial LRC proliferated, suggesting they function as stem/progenitor cells to initiate epithelial regeneration; while in the latter, epithelial LRC and non-LRC rapidly proliferated to regenerate LE and glandular epithelium [80]. Using a mouse model with menstrual breakdown and repair, ERα negative glandular epithelial LRC contributed to the repair of the LE following menstruation, post progesterone withdrawal [81]. Endometrial repair occurred in the absence of oestrogen [81]. BrdU^+^ epithelia was lost soon into the chase period, leading to the thoughts that the epithelial regeneration could be relying on the self-duplication of a mature epithelial cell type, or that the LRC technique is not sensitive enough to label rare endometrial epithelial cells with an ASC phenotype [82].

#### 3.4.2. Stromal LRCs

Between 6–9% of the stroma were LRCs and they were located just below the LE at the endo-myometrial junctions or near the blood vessels [80,82], with 84% of them being ERα negative. These cells were found to not be leucocytes (by excluding CD45 staining), or endothelia (CD31 staining) [80]. BrdU^+^ cells surrounding blood vessels were positive for α-SMA, making it probable that these cells represent pericytes. In some studies, 0.6% of stromal LRCs co-expressed OCT4 (a pluripotency marker) and c-kit (a haemopoietic stem cell marker) [82], so, they were potentially in an undifferentiated state; but in others, neither Sca-1 [80] nor c-kit [83] were expressed. However, to date, there are no studies describing cells co-expressing the classical triad of OCT4, NANOG and SOX2 confirming pluripotency, in the human endometrium.

Oestrogen was shown to drive epithelial LRC proliferation in juvenile development, but had a minimal role in epithelial and stromal LRC cyclical regeneration, perhaps indicating that adjacent BrdU^−^/ERα^+^ endometrial cells release paracrine factors to mediate an LRC response [81].

## 4. Identifying Markers That Are Expressed by Cells Located in the Postulated Stem Cell Niche (Basalis and PM Glandular Epithelium)

Unlike stromal studies, the studies on epithelial stem cell markers considered the hypothesis that the stem cells should reside in the basalis glands, or in the PM epithelium (Figure 1B). The basalis markers described below (SSEA-1, nuclear SOX9, nuclear β-catenin) were the first basalis markers to be presented as epithelial ASC markers in 2013 [13]. Subsequent work has also suggested N-cadherin to be another basalis marker [84], but the very recent work using *LGR5* in situ hybridisation challenges this hypothesis and proposes the existence of more than one epithelial stem cell niche in the human endometrium [85]. These studies, however, are based on the presumption that human endometrial glandular architectural arrangement is a single blunt-ended tube, which is disputed in 3D reconstruction studies [86]. The Nguyen et al., study, in particular, suggests that cells deeper in the glandular base are more likely to be marking the more primitive cell, i.e., a hierarchical arrangement depending on the cellular location within the presumed single tubular, blind-ended glandular structure [84]. Hence, the localisation of these need to be re-examined with the 3D architectural re-modelling of the endometrial glands to fully appreciate the cellular hierarchical arrangement and stem cell organisation of the human endometrial epithelial compartment. In the stroma, only a limited number of stem cell markers have been examined on the basis of their abundance in the postulated stromal stem cell niche, the perivascular region.

### 4.1. SSEA-1

SSEA-1 is a cell surface glycan, an antigenic epitope, defined as Lewis X carbohydrate, and is expressed by preimplantation mouse embryos, teratocarcinoma stem cells, and mouse ESCs [87,88,89,90]. Its presence signifies cells in an undifferentiated state, as expression is lost during stem cell differentiation.

In the human endometrium, immuno-reactivity to SSEA-1 is specific to epithelia and some leucocytes only [13]. Intensity is significantly greater in the epithelium of the proliferative over the secretory phase, and strongest in the basalis, and basalis-like PM endometrium, when compared with the functionalis epithelium. SSEA-1 enriched cell population has a greater propensity to produce gland-like structures in 3D culture, and also has higher telomerase activity and longer telomere lengths. The function of SSEA-1 in the endometrium remains unknown, but it is postulated to be associated with cell adhesion, migration, and capacity to differentiate [13]. 

Fibroblast growth factor (FGF) and Wnt-1 are both involved in stem cell maintenance and differentiation; work has shown that SSEA-1 possibly functions to bind and modulate these growth factors [91], and when this is supplemented with the prominent expression of SSEA-1 in the basalis epithelium, it is conceivable that these cells are a component of the endometrial-epithelial stem/progenitor cell niche. However, this study has not demonstrated any other stem cell properties of the SSEA1^+^ epithelial cells and their in vivo tissue reconstitution ability is also not yet known. More recent work has described some SSEA1^+^ cells to also be located in the LE, thus the expression is not strictly limited to the basalis [85]. The other criticism of SSEA1^+^ cells being progenitors is their relative abundance in the basalis and in the PM endometrium. ASCs are expected to be rare cells in a given tissue. However, the huge regenerative requirement of the human endometrium may require a greater number of ASCs. Moreover, the SSEA-1^+^ cells may be committed progenitors and more primitive ASCs may be a rarer subpopulation of SSEA1^+^ cells. These possibilities remain to be confirmed in future studies.

### 4.2. SOX9

SOX9 is a Wnt target transcription factor, and thus is located in the nucleus; it was first discovered in patients with campomelic dysplasia [92]. SOX9 expression differentiates cells derived from all three germ layers into a large variety of specialised tissues and organs, with roles in chondrogenesis [93,94], male gonad development [95], neural crest development [96] and in the lower crypt region of the intestinal epithelium [97]. SOX9 expressing cells detected by immunohistochemistry (IHC) in normal human endometrium were found to be present in significantly larger numbers in the proliferative phase of the menstrual cycle when compared with the secretory phase [98]. Following this work, another IHC study described SOX9 expression to be largely confined to the basal epithelial cells throughout the cycle, with significantly greater numbers of epithelial cells expressing nuclear SOX9 in the basalis (46.2–52.3%) over the functionalis (8–12.1%) glands. The PM endometrium demonstrated the highest SOX9 immunostaining out of the pre/PM endometrial samples, with over 75% of PM epithelial cells expressing nuclear SOX9 [13].

This paper concluded that nuclear SOX9 co-localised with SSEA-1 and nuclear β-catenin, suggesting an activated Wnt pathway in the basal glands of premenopausal endometrium, could function by maintaining the SSEA-1^+^ cells in a less-differentiated state, representing the endometrial stem/progenitor cell compartment of the stem cell niche, playing an important role in homing stem cells [13]. It was also concluded that high levels of nuclear SOX9 observed in the PM endometrial epithelial cells may function as a checkpoint to prevent hyperplasia, as loss of SOX9 in the intestinal epithelium leads to hyperplasia [99]. Although nuclear SOX9 may mark a potential primitive cell population in the endometrium, due to the nuclear location, SOX9 is obviously not a suitable marker to isolate these cells for further functional studies. The authors further concluded that nuclear SOX9, containing basalis endometrial epithelial cells, can be isolated for functional studies using the surface marker SSEA-1 [13], and their subsequent in vitro experiments demonstrated these epithelial cells to have high telomerase activity and superior ability to generate endometrial gland-like organoids in 3D culture [13]. As mentioned above for SSEA-1, the number of cells containing nuclear SOX9 in the endometrial epithelium is greater than the expected abundance for an ASC population.

### 4.3. Nuclear β-Catenin

The canonical Wnt/β-catenin signalling pathway is involved in cell fate determination. Wnt signalling pathway activation causes β-catenin to enter the nucleus, where it regulates the transcription of target genes [100]. In the absence of Wnt signalling, β-catenin is dislocated from the nucleus. In the highly regenerative intestinal epithelium, nuclear β-catenin is highly expressed in the stem cell region of the intestinal crypt [97,101]. It functions to maintain organisation of the intestinal epithelial cells and is crucial for maintaining an undifferentiated state, evidenced by diminished activity in differentiated cells. Loss of nuclear β-catenin in the intestinal crypt results in rapid degeneration of epithelial cells [101,102].

In the endometrium, the Wnt/β-catenin pathway has been shown to be active in pre and PM endometrial cells [92] and is implicated in regulating the menstrual cycle, with increased nuclear β-catenin expression seen in proliferating endometrial epithelial cells [103,104,105].

Nuclear β-catenin has been identified in a sub population of epithelial cells in the basalis layer of the endometrium co-expressed with SSEA-1 and SOX9, where the stem cell niche is postulated to reside [13]. In this study, nuclear β-catenin expression was restricted to occasional basal glandular epithelial cells, and a similar expression pattern to co-localised SOX9 and β-catenin expression in the intestine was also demonstrated in the endometrium.

Although the published literature on nuclear β-catenin in the endometrium is limited, it does suggest that the endometrial epithelial cells in the basalis which express it could have stem cell properties. This data is in keeping with the current understanding of stem cell activation in the intestinal epithelium.

### 4.4. N-Cadherin

Data from a Wnt-associated gene profiling study of the endometrium [92] identified N-cadherin (gene *CHD2*) to be enriched in the postulated stem/progenitor cell rich postmenopausal endometrium. Given the known importance of Wnt signalling in stem cell biology, the authors investigated the cell surface marker N-cadherin to determine if it could be a potential marker of the endometrial epithelial progenitors [84]. N-cadherin protein was shown to be expressed in 16.7% (range 3.7–36.7%) of epithelial cell adhesion molecule (EpCAM)^+^ endometrial epithelial cells sorted with FACS, and in 20.2% (range 8–35.5%) of the epithelial cells sorted with magnetic beads. When colony forming assays were utilised to assess the enrichment of epithelial progenitors, larger clones and significantly higher median cloning efficiency were observed in the N-cadherin^+^ cells. These clones were large and densely packed, with small cytokeratin-positive cells and a high nuclear:cytoplasmic ratio. When serial cloning was undertaken, N-cadherin^+^ cells generated clones from freshly isolated suspensions and samples underwent up to three rounds of serial cloning, and were also differentiated into cytokeratin^+^ gland-like epithelial structures with a lumen in 3D Matrigel. N-cadherin was found by IF to be strongest in the basalis glands adjacent to the myometrium, and rarely co-localised with Ki-67, indicating a quiescent phenotype. Although some overlap of expression was seen, SSEA-1^+^ cells were described to be phenotypically distinct from N-cadherin^+^ cells, suggesting a potential epithelial hierarchy [84]. Importantly, the authors reported no N-cadherin expression in the LE. Two further IHC studies were also published in the same year. The first compared infertile patients with fibroids, to fertile controls, using IHC and qRT-PCR [106,107]. This showed that N-cadherin was lower in the LE in the mid secretory endometrium of infertile women when compared to fertile controls, but no significant change was demonstrated in either the immuno-expression or the mRNA. The IHC staining demonstrated by Makker et al (LE expresses strongest levels) [106] is in stark contrast to the IF staining presented in the Nguyen et al study (basalis adjacent to the myometrium expresses strongest levels) [84].

Therefore, the published studies on endometrial N-cadherin seem to report major conflicting differences. However, these studies have reported on N-cadherin expression in a variety of patient populations (healthy and pathological) using different techniques; the exact clones identified by different anti-N-cadherin antibodies were inconsistent and this makes it difficult to draw conclusions from the available endometrial N-cadherin data. However, the cells expressing the N-cadherin epitope described by Nguyen et al may have some progenitor activity, and their exact position in the human endometrial epithelial differentiation hierarchy is yet to be confirmed in a functional study and in the context of the recently proposed endometrial epithelial 3D architecture [86]. Finally, similar to SSEA1, the N-cadherin expressing cells are also present in a greater number than what is expected for an ASC population.

## 5. Examining Endometrium for the Expression of Putative Stem Cell Markers That Were Identified to be Expressed in the Epithelial Stem Cells (ESCs), or Stem Cells of Different Tissues (e.g., *OCT4, Mushashi-1, LGR5,* Notch1/numb)

### 5.1. OCT-4

OCT-4 has been proposed as a marker of pluripotent human ESCs and some ASCs. Although the expression of OCT-4 has regularly been associated with primitive cell types by many authors, it is important to appreciate that the synergistic expression of the classical pluripotency gene triad, *OCT-4*, *NANOG* and *SOX2* is required to maintain pluripotency. OCT-4 was seen in some endometrial samples by IHC, and in all endometrial samples by reverse transcriptase-polymerase chain reaction (RT-PCR) with variable expression in the human endometrium [108]. Close scrutiny of the pictures presented in this manuscript reveal that OCT-4 is not expressed in the epithelial cells, but appears to be seen rarely in some stromal cells or blood vessels. The micrographs of the immuno-staining were not supported by further confirmatory data using a secondary method. The authors simply concluded that OCT-4 staining is present and is mostly expressed in the stromal compartment [108]. In a subsequent study, OCT-4 was found not to be differentially expressed during the menstrual cycle in women and is, therefore, proposed to be uninfluenced by hormones [109]. OCT-4 has also been located in some mouse LRCs in the deeper endometrial stroma, co-localising with c-KIT [82]. Therefore, further work is needed (using a reliable antibody, specific to human OCT4a antigen) to examine the cell-specific expression in the human endometrium, since OCT4a is the particular antigen associated with an undifferentiated phenotype. Therefore, we can conclude that in the present time, there is no robust evidence to suggest OCT-4 expressing cells to be relevant to the endometrial ASC population.

### 5.2. Musashi-1

Musashi-1 is an RNA-binding protein in neural stem cells and an intra-cellular epithelial progenitor cell marker that regulates self-renewal signalling pathways. The protein is expected to assume an intracellular location, and thus will not be suitable for use in isolation of the cells that express it for functional studies. Musashi-1 was immunolocalised to single epithelial cells, and small clusters of stromal cells in human endometrium [110]. The authors describe the staining as nuclear and cytoplasmic, but the representative figures presented in the manuscript only demonstrated cytoplasmic staining. IF images showed Musashi-1 to be co-localised with its molecular target, Notch1, and telomerase. Musashi-1 positive cells were mainly found in the basalis in the proliferative stage of the menstrual cycle (when compared to the secretory stage), suggesting their possible stem/progenitor cell function. Stromal Musashi-1 positive cells were not found in a perivascular location, although some were in a peri-glandular region, a similar location to some stromal LRC in mouse endometrium [80].

More recently, Musashi-1 expression has been found in the neonatal endometrium from the 12th week of gestation, with the number of positive cells decreasing with increasing gestational age. In the reproductive endometrium, Musashi-1 staining was seen in dispersed single cells and in stromal cell groups adjacent to myometrium [111].

In summary, there is only limited data on cytoplasmic and nuclear IHC staining for Musashi-1 in the endometrium, without any functional studies confirming the stem cell properties of these cells. To date, no further confirmatory work has been undertaken since the preliminary publication in 2008. Therefore, Musashi-1 is yet to be proven as an endometrial ASC marker.

### 5.3. Notch1/Numb

The family of Notch proteins are ligand-dependent transmembrane receptors that transduce extracellular signals responsible for cell fate and differentiation in a multitude of cellular systems and niches [112,113,114,115]. Notch1 is a heterodimeric, 300-kDa type 1 transmembrane receptor which mediates signaling induced by cell-to-cell contact [116]. Numb is an inhibitory regulator of Notch1 signaling that acts by promoting the ubiquitination and degradation of the Notch1 intracellular domain [114].

Positive Notch1 immuno-staining has been found to be concentrated in the cytoplasm of endometrial epithelial cells, whereas very weak staining has been observed in the stromal cells. This immuno-expression was dynamic in the endometrium, with higher Notch1 in the proliferative phase than in the secretory phase in some studies [114,117], although the reverse was reported in others [115], while no significant difference was observed between the proliferative phase in pre-menopausal and postmenopausal samples. Interestingly, the maximal staining intensity was seen in the mid secretory receptive phase of the menstrual cycle [118]. Strong cytoplasmic immunostaining for Numb was limited to the epithelial cells and very weak staining was observed in the stroma. Contrasting results have been reported, suggesting either consistent levels across the menstrual cycle [114] or decreased immuno-expression in the mid secretory phase [118]. These studies only utilized single technique (IHC) without any secondary confirmatory techniques or functional work [114].

The studies that considered Notch1 as a stem cell marker (showing maintenance of cells in an undifferentiated state) used both IHC and qPCR to confirm the presence of Notch1 in endometrial biopsies, and the clones containing Notch1 were able to differentiate into multiple lineages [119]. However, due to their intracellular location, Notch1/Numb are not suitable markers for isolating the potential ASCs for further study.

### 5.4. MSCA-1

MSCA-1, a bone marrow-derived MSC surface marker, has been identified to be identical to Tissue Non-specific Alkaline Phosphatase (TNAP) [120,121,122,123,124,125]. When ESC’s differentiate, the expression of TNAP decreases [42]. TNAP is expressed on endometrial perivascular cells, the proposed location of endometrial MSC-like cells [42]. The proportion of W8B2^+^CD146^+^ endometrial stromal cells was compared to the proportion of CD146^+^PDGFRβ^+^ MSC-like cells found in the human endometrium, and these were very similar, leading to the conclusion that endometrial MSC-like cells express TNAP. Combined with CD146, this ectoenzyme was proposed to be a suitable marker for the isolation from the EpCAM^−^ endometrial stromal population. Due to TNAP being exclusively expressed in the CD146^+^ subset, but not on other MSC-like/fibroblast-like cells, it would appear that TNAP is developmentally expressed on MSC/pericyte progenitor cells and is down-regulated during further differentiation.

Not only was TNAP expressed on the endometrial perivascular cells, but immuno-staining was detected on endometrial epithelial cells at the apical luminal surface [25]. This has led to TNAP also being proposed as a marker for the isolation of a subset of endometrial glandular epithelial cells. However, the assessment of the ability of TNAP expressing cells to recapitulate endometrial tissue in animal models, and their multilineage potential, would require these cells to be sorted on the basis of their expression of TNAP, as well as EPCAM. Therefore, TNAP may not suitable as a single marker isolation protocol for endometrial MSC. However, the fact that TNAP is expressed in the cells of stromal and epithelial compartments is interesting, and if further studies demonstrate that cells expressing TNAP from both fractions possess stem cell characteristics, it may be a common marker for cells involved in the endometrial regeneration process, and a good therapeutic target. Consequently, further studies are warranted in this area.

### 5.5. LGR5

Leucine-rich repeat-containing G-protein-coupled receptor 5 (LGR5) is a transmembrane receptor [126] characterised by a large leucine-rich extracellular domain [126], belonging to a family of glycoprotein hormone receptors [127]. Little was known about mammalian LGR5 before 2007 [128] when it was discovered by researchers seeking an intestinal stem cell marker [129]. Subsequently, in the human endometrium, by using RT-PCR, *LGR5* gene was found to be expressed in 26 full thickness mid proliferative to late secretory phase samples [130]. Substantial differences were discovered in expression levels of individual women, but no variation was observed throughout the menstrual cycle, therefore suggesting it was not hormonally regulated. This study was followed by a mouse study demonstrating that: murine *lgr5* gene is dynamically regulated in endometrial epithelium expressed only in immature and ovariectomised mice, and is down-regulated by oestrogen. All of this evidence alludes to a hormonal regulation, and for *LGR5* to be lost with differentiation (such as acquiring hormonal responsiveness, refer to Figure 2) [127]. More recently, a review paper proposed LGR5 to be a potential stem cell marker in the human endometrium. The authors also included original data in this review, using IHC to show a population of stromal and epithelial cells stained by an anti-human LGR5 antibody and LGR5^+^ cells mainly located in the perivascular regions [131]. In this review, further original data from telomapping (a type of confocal quantitative fluorescence in situ hybridisation that displays the gradient of telomere length in a given adult tissue) to identify cells with the longest telomeres was also included. The authors subsequently claimed that *LGR5* mRNA signal was present in some of the cells containing the longest telomeres, suggesting that LGR5 expressing cells are associated with a long telomere phenotype. Notably, this statement was made without supporting data. No menstrual cycle differences were reported with regards to the expression of LGR5 protein. The authors concluded that perhaps LGR5 could be considered as a universal stem cell marker and possibly a marker of ASCs in the human endometrium [131]. Although this review presented some limited original work on *LGR5* mRNA and protein, without the provision of detailed methodology (e.g., information on the exact antibody used) [132], scientific scrutiny is not possible for the robustness of that data. Since the specificity of the available anti-human LGR5 antibodies are in considerable doubt, the claim that LGR5-expressing cells identified by IHC are ASCs needs further investigation using more suitable methods. The subsequent publication from the same group described xenografting the isolated LGR5^+^ cells using an anti-human LGR5 antibody into a mouse model [132] to determine their functional relevance. However, the location of the LGR5^+^ cells in the intact human endometrium using the same anti-human LGR5 antibody was not described. Whether this study used the same antibody that they had used in their previous review is also unclear [131]. Human LGR5^+^ epithelial and stromal cells from endometrial biopsies (not full thickness endometrium, and thus will contain only the functionalis layer including LE) were sorted, according to their surface expression of LGR5, using an antibody (of unconfirmed specificity) by FACs, and were phenotypically characterised by flow cytometry with haematopoietic and mesenchymal markers. These LGR5-enriched cells were labelled and injected under the kidney capsule of immunocompromised mice. The authors reported LGR5^+^ cells in the human endometrium to constitute 1.08 ± 0.73%, and 0.82 ± 0.76% of the total cells in the epithelial and stromal compartments respectively. LGR5-enriched cells showed an abundant expression of CD45 (a mature leucocyte marker) and no expression of more primitive surface markers CD31, CD34, CD133, CD73, and CD90. However, co-expression of LGR5^+^ with the macrophage marker CD163 was detected. The tissue recapitulation resulted in a weak endometrial reconstitution, and transcriptomic profiling revealed new attributes for LGR5^+^ cells related to their putative hematopoietic origin. Authors concluded that LGR5 was unlikely to be a universal stem cell marker [132], opposing their previous proposal [131]. They further stated that LGR5^+^ cells appeared to be recruited from blood to be part of the stem cell niche at the perivascular microenvironment, to activate the endogenous niche [132].

When considering the available evidence, the mouse lgr5 studies may not translate well to humans due to obvious species-specific differences in their endometrial biology. The initial study examining human *LGR5* mRNA level did not attempt to ascertain the location of the endometrial *LGR5* expression [131]. The specificity of all presently available anti-human LGR5 antibodies to identify the protein are not confirmed, and are of considerable doubt [133].

To overcome the above deficiencies in the literature, earlier this year, the gold standard method of in situ hybridisation (ISH) was utilised alongside qRT-PCR, IHC and in silico analysis of published endometrial microarray datasets to conclusively examine the cellular location of LGR5 expression in full thickness normal human endometrium [85]. *LGR5* expression was limited to the epithelial compartment of the endometrium, with high *LGR5* expressing cells seen in the endometrial LE and in the stratum basalis; the LE expressed significantly higher levels of *LGR5* than all other epithelial compartments. The dynamic spatiotemporal pattern of *LGR5* expression suggested hormonal regulation, with a reduction in *LGR5* expression in the secretory phase (with ISH) in the luminal and functionalis epithelium respectively. Endogenous and exogenous progestogens inhibited *LGR5* expression in the endometrium both in vivo and in vitro in explant culture. When endometrial samples of women taking synthetic progestogen treatment (progesterone only pill, ‘POP’, or levonorgestrel-releasing intrauterine system, ‘LNG-IUS’) were compared with the samples of women not on any treatment, a significant reduction of *LGR5* mRNA levels was observed. Data was further confirmed by analysing previously published microarray datasets. The epithelial compartment-specific expression pattern of LGR5 in the full thickness endometrium prompted the novel theory that more than one epithelial stem/progenitor cell pool could exist in the human endometrium; one residing in the basalis (SSEA-1^++^/SOX9^++^/*LGR5*^+^) supporting the massive regeneration of the functionalis after menstrual shedding or parturition; while the other (*LGR5^++^/*SSEA-1^+^/SOX9^+^) supports the embryo-implantation process, and maintains the LE cells that are likely to be lost on a daily basis [85]. However, this study was an observational study without any functional data. It is therefore important in the future, when a reliable anti-human LGR5 antibody is available, for further work to be carried out to assess the LGR5-enriched cells from the endometrium for stem cell function.

### 5.6. Telomerase

Telomerase is an RNA-dependent DNA polymerase enzyme responsible for synthesising and maintaining telomeres that exists at the ends of all linear chromosomes (Reviewed in Hapangama et al., 2017 [134]). The expression of telomerase in human cells is essential for maintaining cellular integrity and immortalization. TERT is the catalytic subunit of telomerase holo-enzyme, and has been shown to be expressed in cells with self-renewing potential, including stem cells [135]. TERT^+^ intestinal epithelial cells are considered to be intestinal stem cells which are quiescent and are regenerated in response to tissue injury [136].

In the human endometrium, telomerase activity is limited mainly to the glandular epithelium [137]. Isolated SSEA-1^+^ basalis progenitor epithelial cells grown in culture had a significantly higher telomerase activity, longer mean telomere lengths and the ability to generate endometrial gland-like structures than SSEA-1 depleted epithelial cells [13]. This study suggests that high telomerase activity in SSEA-1^+^ epithelial cells may render this epithelial progenitor population to have an increased replicative lifespan and possibly self-renewal, which are accepted stem cell properties. 

Further evidence suggests telomerase activity to mark potential endometrial stem cells, comes from a study where an increased number of epithelial cells co-expressed the ASC marker Mushashi-1 and telomerase reverse transcriptase (TERT) in the proliferative phase endometrial samples when compared with normal secretory endometrium [110]. The reliability of anti-human telomerase antibodies is known to be problematic; therefore, caution should be taken considering this antibody-based telomerase study.

In a transgenic, green fluorescent protein (GFP) reporter mouse model, a small population of *mTERT*^+^ presumed ASCs were identified in the endometrial luminal and glandular epithelial cells [138]. *mTERT*^+^ cells decreased in response to ovariectomy of the mouse, suggesting a role of ovarian steroid hormones in maintaining these cells. In other tissues such as bone marrow [139], *mTERT*-expressing cells are considered to possess ASC qualities. The study, however, did not assess the functional ASC activity of *mTERT^+^* endometrial cells. Adequate telomerase activity is a prerequisite of proliferating endometrial epithelial cells [140]; therefore, although there is evidence for telomerase activity in the proposed ASC compartment in the human endometrium, further work is needed to clarify if telomerase is a specific phenotypical ASC marker or is merely marking the activation status/proliferation of the ASCs. 

## 6. Involvement of Endometrial Stem Cells in Endometrial Proliferative Disease

The involvement of endometrial ASC in proliferative disease such as endometriosis and endometrial cancer has been postulated [15]. Any recurrent or persistent disease of the premenopausal endometrium, from heavy menstrual bleeding, infertility, or recurrent miscarriage should be originating from abnormalities accumulated in the ASC population that is responsible for the regrowth of a novel functionalis layer each month. Although the theoretical possibility that all endometrial pathologies originate from aberrant endometrial ASCs is widely accepted, the direct evidence available supporting this theory is scarce. Therefore, in this review, we have highlighted two endometrial proliferative conditions, endometriosis and endometrial cancer, to highlight some of the interesting data from the endometrial stem cell marker prospective.

### 6.1. Endometriosis

Endometriosis is a common, benign proliferative disease of the endometrium defined as having endometrium-like tissue existing outside of the uterine cavity. One in 10 women of reproductive age in the UK suffer from endometriosis, which is responsible for significant morbidity and places a huge economic burden on the women, health services and society in general. Little is known about its aetiology and pathogenesis and this prevents the formulation of novel treatments [141]. The ectopic endometriotic tissue retains hormone responsiveness and may undergo inflammation, proliferation and regeneration, and thus stem cells are thought to be involved in the pathogenesis. Leyendecker et al. proposed that basalis endometrial cells enriched with progenitor potential are shed with menstruation in women with endometriosis and these cells may give rise to ectopic lesions after retrograde menstruation and trans-tubal migration in to the pelvic cavity [142]. The studies demonstrating the expression of epithelial stem cell markers such as SSEA-1, SOX9 and nuclear β-catenin [13] and Musashi-1 [110] in ectopic endometriotic lesions have suggested a possible direct involvement of ASCs in endometriosis lesion formation. High telomerase activity is a feature of endometriosis [143] and is also found in SSEA-1 expressing basalis progenitor epithelial cells [13]. Furthermore, Musashi-1 expressing endometrial epithelial cells have been shown to co-express telomerase catalytic subunit (hTERT) [110]. The involvement of ASC’s in the pathogenesis of endometriosis is further supported by increased expression of Numb and Notch1 in eutopic endometrium from patients with endometriosis, when compared with controls, and could be associated with increased severity of the condition. Knock down of Notch1 in human endometrial epithelial and stromal cells resulted in reduced cellular proliferation and migration when injected into the peritoneal cavity of mice, and a reduced size of resulting endometriotic lesion was observed, implicating Notch1 in the pathogenesis of endometriosis [113]. An IHC study also reported increased Notch1 expression in adenomyosis. Studies are awaited to determine the presence or the involvement of cells expressing N-cadherin and other endometrial stromal ASC markers in ectopic endometriotic lesions formation. Interestingly, a recent study which analysed synonymous and missense somatic passenger mutations has suggested that ectopic endometriotic lesions contain clonal populations of epithelial cells originating from, presumably, an ectopically situated epithelial ASC, whereas stromal cells may be continuously regenerated or recruited over the course of disease [144,145]. This data presents a novel concept, in that the primary cell type initiating and regulating the initiation as well as persevering the ectopic lesions could be the epithelial ASC and they may subsequently recruit stromal ASCs to create the endometrial niche. Further studies are needed to examine this novel hypothesis. 

### 6.2. Endometrial Cancer

Endometrial cancer is the most common gynaecological cancer with an increasing incidence. In 2015 alone, 8984 new cases of endometrial cancer were diagnosed [146] and in 2016 [146], it was the cause of 2360 deaths in the UK. With estimated increases of over 90% in annual costs for endometrial cancer surgery, this places a huge burden on the NHS and society’s resources. Furthermore, alternative therapy is urgently needed for the recurrent and metastatic disease that is resistant to both chemo and radiotherapy. Specialised cancer cell sub-populations called cancer stem cells (CSC) are postulated to be responsible for distant metastasis, cancer recurrence and resistance to chemo/radio therapy. High telomerase activity is also implicated in cancer metastasis and CSC. CSC share many features with adult tissue ASC in that they express telomerase, have self-renewal capabilities and higher proliferative potential. SSEA-1 and Musashi-1 expressing potential endometrial epithelial ASCs from healthy endometrium have been shown to have telomerase activity [13,110] and the malignant transformation of these ASC is thought to initiate cancer. High levels of Musashi-1 expression has shown to be associated with poor prognosis in endometrial cancer, suggesting that Musashi-1 expressing CSCs are a possible therapeutic target [110,147,148]. SOX9 is also upregulated in endometrial cancer and upregulation of SOX9 is a feature of the premalignant hyper-proliferative condition, endometrial hyperplasia [98,149], suggesting an involvement of the basalis progenitor ASC in these conditions. Using purely IHC, Xie and colleagues compared N-cadherin expression between patients with endometrioid adenocarcinoma, and normal controls [150]. They showed that N-cadherin was positive when brown/yellow particles were seen in the cytoplasm of a cell (again contradictory to other published studies). For the 50 normal samples that were included in this study, the positive expression rate for N-cadherin protein was 40.0% (8 weakly positive, 9 moderately positive, and 3 strongly positive) and the positive N-cadherin protein expression rate was statistically higher in the endometrioid adenocarcinoma group compared to the normal controls. They also showed that E-cadherin is not commonly expressed in the N-cadherin expressing cells, therefore, a transition may exist between them [150]. An increase in Notch1 expression was reported in endometrial cancer samples [115] in a study using IHC. Stromal Notch1 expression increased in endometrial carcinoma with respect to hyperplasia and polyps. The cell fate determinant Numb, has also been reported to be increased in endometrial cancer, compared to normal endometrium in a study using IHC [151], and the immune-staining gradually increased in correlation with the advancing grade of the endometrial cancer samples. However, functional studies are needed to examine the role of Notch1 and Numb in endometrial carcinogenesis. However, there are no studies to date examining the expression or the involvement of the other proposed normal endometrial ASC markers in endometrial cancer.

## 7. Conclusions

The human endometrium obviously contains ASCs that are responsible for its frequent, efficient and scar-less regeneration. Recent work suggests that the main endometrial cell lineages, epithelium and stroma, may develop independently; and neither cell types are likely to originate from bone marrow-derived cells. There are many stromal and epithelial ASC markers proposed, with some demonstrating in vitro stem cell properties, yet the in vivo tissue reconstitution ability of these cells has either been poor or not yet fully examined. A summary of postulated endometrial stem cell markers in the epithelia, stroma, and perivascular cell populations can be seen in Table 1. The differentiation potential of the stromal and epithelial cells (isolated to ascertain their therapeutic utility) needs to be fully confirmed in the future. The demonstration that there are epithelial cells expressing the described ASC markers in endometrial proliferative conditions supports their involvement in the pathogenesis of endometriosis and endometrial cancer, yet further studies are needed to ascertain the possibility of targeting them for curative therapy.

## Figures and Tables

**Table 1:** Summary of postulated endometrial stem cell markers in epithelial, stroma, and perivascular cell populations.

Cell Type	Location	CD146/PDGFRβ	SUSD2	SP	LRCs	SSEA-1	SOX9	Nuclear β-Catenin	N-Cadherin	OCT4	Musashi-1	Notch/Numb	MSCA-1	LGR5	Telomerase
**Epithelial**	Luminal	−	−	?	+	+	+	−	−			+	+	++	
	Functionalis	−	−	?		−	−	−	−		+	+	−	−	+
	Basalis	−	−	?		++	++	+	++		+	+	−	+	+
	Undefined			+											
**Stromal**	Functionalis				+										
	Basalis				+										
	Undefined			+						+	+				
**Peri-vascular**	Functionalis	+	+		+						?				
	Basalis	+	+		+								+		
**References**		[22,42,43]	[15,44]	[1,45,47,48,49,50,51,52,53]	[2,80,81,82]	[13,85,87,88,89,90,91]	[13,92,93,94,95,96,97,98,99]	[13,97,100,101,102,103,104,105]	[84,92,106,107]	[82,108,109]	[80,110,111]	[112,113,114,115,116,117,118,119]	[25,42,112,120,121,122,123,124]	[85,126,127,128,129,130,131,132,133]	[13,110,134,135,136,137,138,139]

Key: ++ strongly positive, + positive, − negative, ? existing evidence unclear.
